# Supplementary material for: Hunting Drosophila viruses from wild populations: A novel isolation approach and characterisation of viruses
Source: PLoS Pathog. 2023 Mar 30;19(3):e1010883. doi: 10.1371/journal.ppat.1010883 (PMC10109509; doi:10.1371/journal.ppat.1010883)
Supplement: S1 Table — (PDF) [file ppat.1010883.s001.pdf]

Table S1. Virus detected by qPCR in 19 samples showing high cytopathic effect and high dsRNA concentration

Sample ID	Place	Country	Host	N flies	Sex	Virus
gb-08052019-2-26	Gialousa	Cyprus	*D. simulans*	5	females	DCV
gb-09052019-3-59	Plantation	Cyprus	*D. simulans*	5	females	LJV
gb-09052019-3-60	Plantation	Cyprus	*D. simulans*	5	females	LJV
gb-08052019-5-91	Gialousa	Cyprus	*D. simulans*	5	females	LJV
gb-08052019-5-93	Gialousa	Cyprus	*D. simulans*	5	females	
gb-08052019-5-98	Gialousa	Cyprus	*D. simulans*	5	males	LJV
gb-10052019-2-112	Gialousas	Cyprus	*D. repleta*	5	females	
gb10052019-3-126	Gialousas	Cyprus	*D. simulans*	5	females	
gb10052019-3-127	Gialousas	Cyprus	*D. simulans*	5	females	
gb-11052019-3-130	Gialousas	Cyprus	*D. immigrant*	6	females	
gb-11052019-2-131	Gialousas	Cyprus	*D. immigrant*	5	females	DCV
gb15052019-4-305	Gialousa	Cyprus	*D. melanogaster*	7	males	LJV
gb15052019-4-306	Gialousa	Cyprus	*D. simulans*	7	males	LJV
GOY-2105209-1-311	Granada	Spain	*D. virilis*	4	females	LJV
gb-09102019-1-386	Cambridge	UK	*D. melanogaster*	10	males	NFV, LJV
gb-09102019-1-390	Cambridge	UK	*D. melanogaster*	5	males	
gb-09102019-1-391	Cambridge	UK	*D. melanogaster*	5	males	LJV
gb-09102019-2-392	Cambridge	UK	*D. melanogaster*	5	females	NFV, Kallithea
gb-09102019-1-393	Cambridge	UK	*D. melanogaster*	7	females	NFV, Kallithea
