# Supplementary material for: Hunting Drosophila viruses from wild populations: A novel isolation approach and characterisation of viruses
Source: PLoS Pathog. 2023 Mar 30;19(3):e1010883. doi: 10.1371/journal.ppat.1010883 (PMC10109509; doi:10.1371/journal.ppat.1010883)
Supplement: S2 Table — (PDF) [file ppat.1010883.s002.pdf]

Table S2. Accession and Isolate Numbers of Virus Sequences

Virus	Name on Tree	Isolate_or_Accession_Number
LJV	D_mel_Cyprus	OP263079
LJV	D_repleta_Spain_Granada	OP263078
LJV	D_sim_Cyprus_1	OP263080
LJV	D_sim_Cyprus_2	OP263081
LJV	Drosophilidae_France_Montpellier	KP714073
LJV	Drosophilidae_Ghana_Accra_1	KP714074
LJV	D_mel_Ghana_Accra_1	KP969709
LJV	D_mel_France_Montpellier_1	KP969710
LJV	D_mel_USA_San_Diego_CA_1	KP96971
LJV	D_sim_USA_Athens_GA_1	KP969712
LJV	D_mel_USA_Athens_GA_1	KP969720
LJV	D_mel_USA_Ithaca_NY_1	KP96972
LJV	D_mel_USA_Athens_GA_2	KP969722
LJV	D_mel_USA_San_Diego_CA_2	KP969724
LJV	D_mel_USA_Athens_GA_3	KP969725
LJV	D_sim_Australia_Torquay	KP969726
LJV	D_sim_Greece_Athens_1	KP969727
LJV	D_mel_France_Montpellier_2	KP969728
LJV	D_mel_USA_Ithaca_NY_2	KP969729
LJV	D_mel_Morocco_Marrakesh	KP969730
LJV	D_mel_USA_Ithaca_NY_3	KP96973
LJV	D_sim_Greece_Athens_2	KP969732
LJV	D_mel_France_Montpellier_3	KP969733
LJV	Drosophilidae_France_Marseille	KP969734
LJV	D_mel_France_Montpellier_4	KP969735
LJV	D_mel_USA_Ithaca_NY_4	KP969736
LJV	D_sim_USA_Athens_GA_2	KP969737
LJV	D_mel_Portugal_Lisbon_1	KP969738
LJV	D_mel_Portugal_Lisbon_2	KP969740
LJV	D_sim_Greece_Athens_3	KP96974
LJV	D_mel_USA_Athens_GA_4	KP969742
LJV	D_mel_USA_Athens_GA_5	KP969743
LJV	D_mel_USA_Athens_GA_6	KP969744
LJV	D_mel_USA_Athens_GA_7	KP969745
LJV	D_sim_USA_Athens_GA_3	KP969746
LJV	D_sim_USA_Athens_GA_4	KP969747
LJV	D_mel_USA_Athens_GA_8	KP969748
LJV	D_sim_USA_Athens_GA_5	KP969749
LJV	D_mel_USA_Athens_GA_9	KP969750
LJV	D_mel_USA_Ithaca_NY_5	KP96975
LJV	D_mel_Greece_Athens_1	KP969752
LJV	D_mel_Greece_Athens_2	KP969753
LJV	D_mel_USA_Athens_GA_10	KP969754
LJV	Drosophila_Portugal_Lisbon	KP969755
LJV	D_mel_USA_Athens_GA_11	KP969756
LJV	D_mel_USA_Athens_GA_12	KP969757
LJV	D_sim_USA_Athens_GA_6	KP969758
LJV	D_mel_USA_Athens_GA_13	KP969759
LJV	D_mel_Portugal_Lisbon_3	KP969760
LJV	D_sim_France_Marseille	KP96976
LJV	D_sim_Greece_Athens_4	KP969762
LJV	D_mel_Ghana_Accra_2	KP969763
LJV	D_mel_USA_San_Diego_CA_3	KP969764
LJV	D_mel_Ghana_Accra_3	KP969765
LJV	D_mel_Ghana_Accra_4	KP969766
LJV	D_mel_France_Montpellier_5	KP969767
LJV	D_mel_Ghana_Accra_5	KP969768
LJV	D_mel_Ghana_Accra_6	KP969769
LJV	D_mel_Australia_Coffs_Harbour	MH384268
LJV	D_mel_Australia_Hunter_Valley_1	MH384278
LJV	D_mel_Australia_Hunter_Valley_2	MH384285
LJV	D_mel_Australia_Melbourne_1	MH384312
LJV	D_mel_Australia_Melbourne_2	MH384324
LJV	D_mel_Australia_Melbourne_3	MH384332
LJV	D_mel_Australia_Melbourne_4	MH384357
LJV	D_mel_Australia_Melbourne_5	MH384368
LJV	D_mel_Australia_Melbourne_6	MH384384
LJV	Apis_mellifera_Ethiopia_Ganta_Afeshum	MT681680
LJV	D_suzukii_Germany	MW556743
LJV	D_sim_Australia_Raeburn_Orchards	MW976818
LJV	Drosophilidae_Ghana_Accra_2	NC027128
LJV	Environmental_Sample_Slovenia	OL472185
NFV	D.mel_Cambridge UK 1	OP263083
NFV	D.mel_Cambridge UK 2	OP263082
NFV	D.mel_Cambridge UK 3	OP263084
NFV	Drosophilidae	KP714070
NFV	D.mel_Maryborough Australia 1	MH384302
NFV	D.mel_Maryborough Australia 2	MH384307
