# Supplementary material for: Hunting Drosophila viruses from wild populations: A novel isolation approach and characterisation of viruses
Source: PLoS Pathog. 2023 Mar 30;19(3):e1010883. doi: 10.1371/journal.ppat.1010883 (PMC10109509; doi:10.1371/journal.ppat.1010883)
Supplement: S1 Fig — (a) DL2-B2 cells overexpressing V5 tagged B2 fusion protein and incubated in the presence of CuSO4 (5 mM) or (b) uninduced (CuSO4-free) for 24 h. Cells were stained with V5 tag mouse monoclonal antibody (1:1000) and labeled with Alexa Fluor 488 rabbit anti-mouse IgG secondary antibody (1,1000). Nuclei (blue) was stained using Hoechst. (PDF) [file ppat.1010883.s003.pdf]

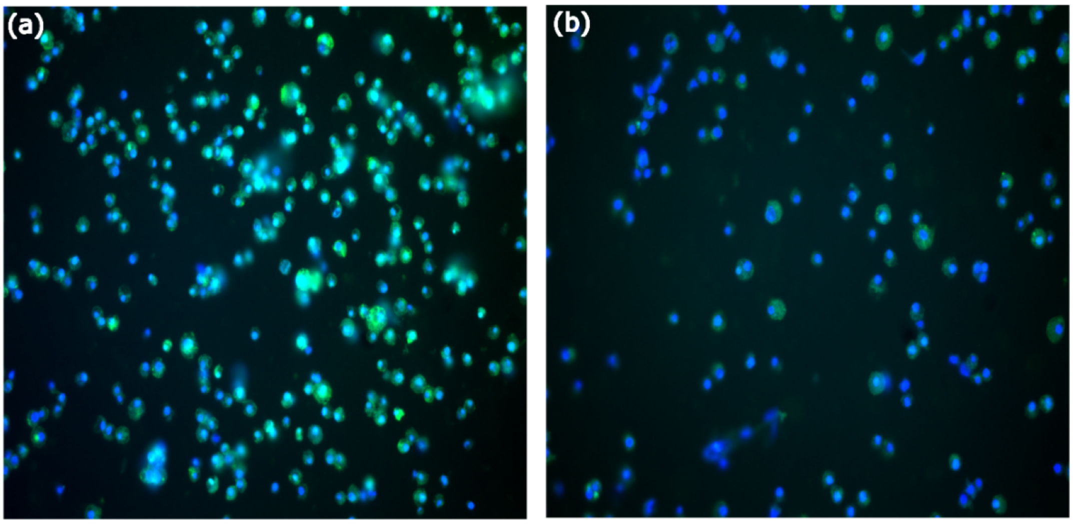

Figure S1. Immunofluorescent images showing the expression of the plasmid.
(a) DL2-B2 cells overexpressing V5 tagged B2 fusion protein and incubated in the presence of $CuSO_4$ (5 mM) or (b) uninduced ($CuSO_4$-free) for 24 h. Cells were stained with V5 tag mouse monoclonal antibody (1:1000) and labeled with Alexa Fluor 488 rabbit anti-mouse IgG secondary antibody (1:1000). Nuclei (blue) was stained using Hoechst.
